# RapidArc quality assurance through MapCHECK

**DOI:** 10.1120/jacmp.v12i2.3251

**Published:** 2011-01-05

**Authors:** Aime M. Gloi, Robert E. Buchanan, Corrie L. Zuge, AnnDrea M. Goettler

**Affiliations:** ^1^ Radiation Oncology St Vincent Hospital Green Bay Wisconsin 54307 USA

**Keywords:** RapidArc, partial arc, ion chamber

## Abstract

The purpose is to devise a patient‐specific quality assurance procedure for RapidArc radiotherapy using the MapCHECK detector array. We use our existing MapCHECK system and a Solid Water phantom with an embedded ion chamber to develop a quality assurance procedure for RapidArc treatment after commissioning. The ion chamber used to measure the absolute dose is surrounded by 6 cm layers of solid water on the anterior and posterior sides. Partial arcs derived from the treatment planning system were used with MapCHECK to determine the actual shape of the dose and correct for the angular dependence. The ion chamber measurements were within 1% of the absolute doses predicted by the Eclipse treatment system. When using a partial arc from 60° to 300° on the MapCHECK array (gamma index <1: 3%, 3 mm, 10% threshold), we obtain a 97.52% average passing rate. A combination of ion chamber phantoms, partial arcs and the MapCHECK system can be used for quality assurance of RapidArc therapies.

PACS number: 3.6.96.0

## I. INTRODUCTION

As a supplement to tomotherapy, Yu^(1)^ suggested intensity‐modulated arc therapy (IMAT). Over the years, increasing computer power and the creation of new planning systems, such as the analytical anisotropic algorithm (AAA), have made a new generation of fast radiation therapies possible. One of these, volumetric modulated arc therapy (VMAT), delivers radiation continuously while the gantry of the linear accelerator rotates through one or more arcs. Several parameters are involved in this process: the aperture shape and orientation of the multileaf collimator (MLC), the gantry rotation speed and the dose rate. Furthermore, VMAT requires the linear accelerator controller to establish a real‐time relationship between these parameters. This synchronization is a daunting task, but necessary for efficient delivery. The difficulties associated with VMAT require a specific means of quality assurance for its patients.

RapidArc (Varian Medical Systems, Palo Alto, CA), a novel planning and delivery system for volumetric, intensity‐modulated arcs, is based on the model and method developed by Otto^(2)^. As a form of VMAT, it calls for efficient and accurate methods to verify the calculated dose distributions. Several detector arrays are already on the market for this purpose. ArcCHECK (Sun Nuclear; Melbourne, Florida, USA) is a cylindrical array 21.0 cm in diameter, containing 1386 0.8 mm×0.8 mm detectors. This device displays a “beam's‐eye” view of the dose distribution throughout the delivery process. Another system, the MartiXX, (IBA Dosimetry, Bartlett, TN), is a 2D ionization chamber array. It consists of 1020 air‐vented, plane‐parallel cylindrical ionization chambers 0.45 cm in diameter and 0.5 cm in height. They are arranged in a $24\times 24\;{\text{cm}}^{2}$ area with centers separated by 0.76 cm. The MartiXX array runs with two separate counters to avoid dead time. Its resolution is 0.76 cm, but signals can be interpolated down to 0.1 cm. Our own group possesses the MapCHECK array (Sun Nuclear; Melbourne, Florida, USA). The device has been described elsewhere^(3)^ as follows:

“It consists of 445 n‐type solid state diode detectors. The inner 221 detectors cover the central $10\times 10\;{\text{cm}}^{2}$ and are arranged in a zigzag pattern so that the diagonal spacing between detectors is 0.707 cm. In addition, the outer 224 detectors are arranged in a similar pattern, but with a diagonal spacing between detectors of 1.414 cm. The array covers an area of $22.0\times 22.0\;{\text{cm}}^{2}$. The active detector area of each diode is $0.8\times 0.8\;{\text{mm}}^{2}$.”

To the best of our knowledge, MapCHECK has not been used by any other group to evaluate RapidArc (hereafter RA) quality assurance through a partial arc. The device is a good candidate for arc therapy because of its high diode accuracy, crucial to patient QA. MapCHECK can also measure steep dose gradients in the penumbra region, and provide QA results while measurements are being performed. MapCHECK evaluates both absolute and relative doses. In this paper, we propose a QA procedure for RA using partial arcs that eliminates the need for film and ion chamber dosimetry. More investigations are needed to show that the partial arc derived from an actual plan could be used as QA for arc therapy.

A number of prior tests specific to RapidArc were performed to ensure good performance of the Trilogy system. We investigate the dynamic multileaf collimator by measuring the output at several gantry positions. This test will evaluate the effect of gravity on leaf position. Then two Picket Fence tests with a stationary gantry were performed during a RapidArc treatment to illustrate the effect of gantry rotation on MLC positional accuracy. Additional tests verified the accuracy of dose rate and gantry speed. All these tests were performed using the Varian RA commissioning and QA files and analyzed using the Dynalog (Bloomfield Hills, MI) file viewer.

The aim of this work is to design a suitable quality assurance system using the MapCHECK device for patients undergoing the RA procedure.

## II. MATERIALS AND METHODS

This study is based on two quality parameters: the absolute dose delivered by a given RA plan, and the partial arc.

**Figure 1:**
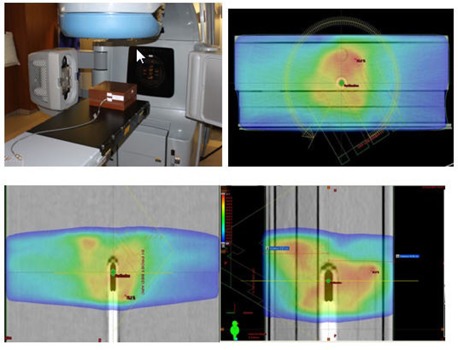
The Trilogy platform, ready with an ion chamber phantom for absolute dose measurement. The upper right and lower panels show dose‐washed images.

First, the absolute dose is determined from the plan using an N30013 Farmer ion chamber (CNMC, Nashville, Tennessee). The ion chamber has a buildup cup and a sensitive volume of 0.6 cc. It is embedded between two 6 cm layers of Solid Water (posterior and anterior) and tomographically scanned using a GE HiSpeed Medical Systems CT‐Simulator (Milwaukee, USA). The dataset gathered from this scan is transferred to the Varian Eclipse (8.6.1) treatment planning system (TPS) (Varian Medical Systems, Palo Alto, CA), where the ion chamber is contoured (Fig. 1). A verification plan or quality assurance plan (i.e., a QA protocol) is then generated and recalculated on the CT image of the ion chamber phantom, in order to compare measured and planned doses. The point of measurement for calculation of the absolute dose is the isocenter of the ion chamber.

To determine the partial arc of the RA plan, we scan a MapCHECK diode array (Model 1175, Sun Nuclear, Melbourne, FL) with 3 cm layer of Solid Water buildup in addition to that inherent to the device.

**Figure 2:**
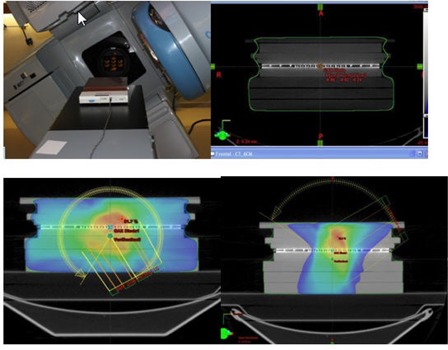
Complete setup for acquisition of a partial arc with MapCHECK. The upper right and lower panels show dose‐washed images

The total amount of material present is equivalent to a 5 cm layer of tissue. Using the same CT dataset, another verification plan is created and applied to the scanned MapCHECK device (Fig. 2). Based on this second plan, two partial arc verification plans are generated: one from 60° to 300°, and another from 300° to 60°. Next, fluence maps of the partial arcs are calculated by the TPS. These maps are used to assess the accuracy of the isodose distribution, and to test for agreement between the measured and calculated relative dose. This analysis provides values for the number of passing points (the number of points where the relative difference in dose and the distance to agreement fall below specified thresholds). Finally, the dose on the central axis of the MapCHECK array is compared to the “expected dose” derived from the fluences generated by the TPS. (The software procedure for creating a partial arc plan is detailed in the Appendix.)

### A. Measurements

The procedure for measuring the absolute dose is as follows. First, the plan is delivered via DICOM transfer to the machine (Trilogy). Next, the absolute dose to the ion chamber is measured as described above. The charges are collected by an electrometer (Model 206), then corrected for pressure and temperature using readings from a digital barometer (Model DB‐705) and thermometer (Model DT4378). All three devices are produced by CNMC (Nashville, Tennessee). Figure 1 illustrates the setup for the absolute dose measurement. The two partial arc plans are also sent to the machine through DICOM transfer. The MapCHECK (Fig. 2) device is placed on the Trilogy table at a source–skin distance (SSD) of 95 cm (including the 5 cm of tissue‐equivalent buildup). The partial arc plans are then delivered in their entirety. RA1 refers to the clockwise (CW) arc from 60°–300°, and RA2 refers to the counterclockwise (CCW) arc from 300°–60°.

### B. Statistical analysis

This section assesses the level of agreement between the expected absolute dose (given by the TPS) and the measured absolute dose using two statistical methods. First, we calculate a concordance correlation coefficient (CC), defined as the product of an accuracy measure (in this case the bias correction factor, $C_{b}$, and a precision measure (in this case the Pearson correlation coefficient, ρ. Thus, for this study we have $\text{CC}=\rho \times C_{b}$. Second, we apply the Bland‐Altman^(4)^ method of assessing agreement. This approach plots the mean of each data pair (the expected and actual doses) against its difference, and defines 95% limits of agreement at the average difference plus or minus 1.96 times the standard deviation of the difference. All statistical calculations were performed using the package Graphpad Prism (GraphPad Software Inc, La Jolla, CA). P‐values less than 0.05 were considered statistically significant.

## III. RESULTS

**Figure 3:**
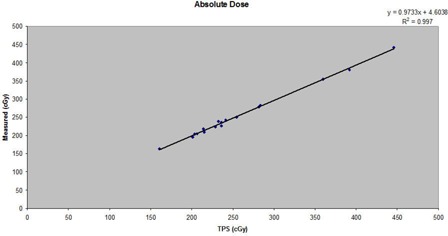
Absolute doses calculated in Eclipse for the TPS compared to absolute doses measured in an ion chamber phantom, for the 17 patients studied.

We analyzed the CT scans of seventeen patients. Two were pelvic scans, and the other fifteen were for patients with prostate cancer. The axial, sagittal and coronal dose‐washed distributions of one subject are shown in Fig. 1. Table 1 shows the expected (TPS) and measured absolute doses acquired for each subject's treatment plan, as well as means and standard deviations. In addition, we recorded the central axis dose through MapCHECK for all 17 patients. Figure 3 compares the expected and measured point doses for each subject. The plot reveals a strong correlation (Pearson correlation coefficient=0.997,p=0.001) that could be seen as demonstrating the validity of traditional QA guidelines for treatment plans. Regression analysis between the two variables confirms a strong linear relationship. Table 2 summarizes the statistical tests comparing the expected and measured absolute doses. The CC coefficient is 0.9978 (95% CI: 0.9941 to 0.9992). This value is based on the correlation coefficient ρ of 0.997 and a bias correction factor $C_{b}$ of 0.992. The latter value simply shows that the slope of the best‐fit line is close to unity.

**Table 1:** The differences between expected absolute doses (predicted by the TPS) and absolute doses measured in our ion chamber phantom (second and third columns) for each patient's treatment plan. The shapes of the dose distributions were verified based on the number of diodes satisfying MapCHECK's gamma criterion (fourth column). Finally, we compare the MapCHECK dose on the central axis to its predicted value (fifth and sixth columns). Summary statistics of these measures are given in the bottom rows.

	*Absolute Dose*			*Mapcheck*	
*Patient*	*Expected*	*Measured*	*Passing*	*Measured*	*Expected*
1	228.9	224.2	100	78.3	75.85
2	235.8	235.8	100	92.33	97.45
3	206.2	205.4	97.9	121.81	121.62
4	391.5	380.5	98.5	124.45	124.75
5	232.6	238	96.9	158.78	161.95
6	254.6	250.4	99.5	119.87	122.52
7	241.1	242.3	95.2	92.86	76.1
8	200.7	195.4	96	82.86	75.42
9	214	217.9	97	82.98	81.27
10	215.5	209.3	96.3	48.1	44.96
11	160.4	163.1	95.8	92.7	93.73
12	203.2	204.3	97.6	108.21	108.43
13	283.6	282.3	99.7	284.35	297.97
14	281.8	279.4	95.5	80.26	78.99
15	236.2	227.1	97.2	127.1	113.93
16	359.6	354.7	96.3	45.01	32.85
17	445.9	442.4	98.5	75.86	73.21
MEAN	258.3294	256.0294	97.52353	106.8135	104.7647
S.D	72.62554	70.79058	1.562626	52.48433	57.05988

**Table 2:** Intraclass correlation (ICC) analysis of the expected and measured absolute doses.

*Parameters*	*Results*
N	17
Concordance correlation Coefficient (CCC)	0.9977
95 C.I	0.9942 to 0.991
Pearson ρ (precision)	0.9985
Bias correction factor $C_{b}$ (Accuracy)	0.9992

**Figure 4:**
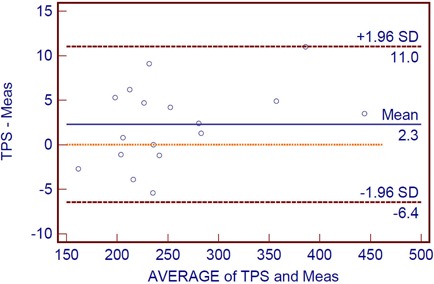
Bland‐Altman plot for the calculated absolute doses and measured doses. The outer lines indicate the 95% limits of agreement (−6.4, 11), and the center line shows the average difference (2.3). Below the center line, the dotted line indicates zero difference between the two doses.

**Figure 5:**
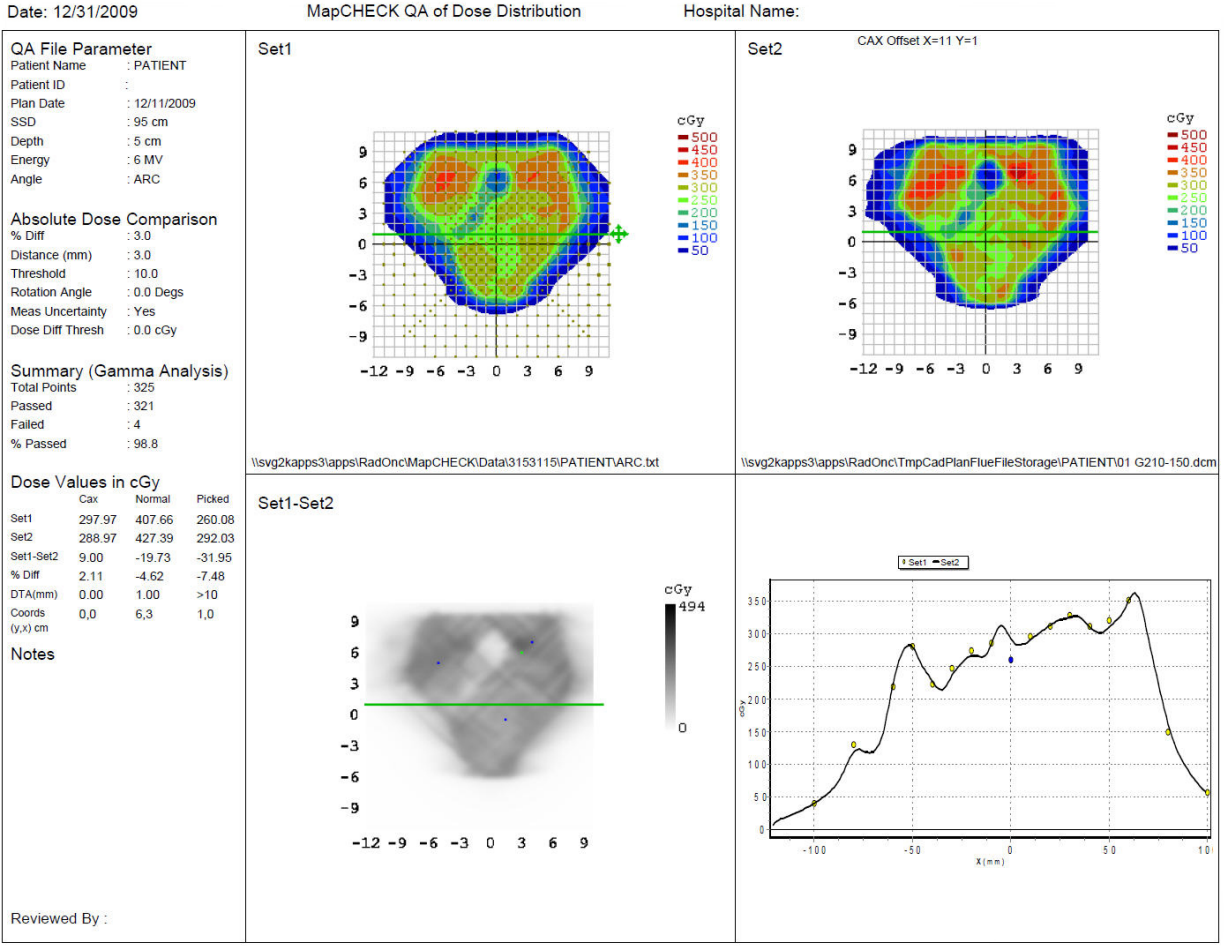
Dose profile of a pelvis measured via MapCHECK using the partial arc method. The upper left represents the measured dose, and the upper panel shows the fluence through Eclispe TPS. The lower left panel compares the profiles, and assesses the gamma criterion (less than 3% difference between the measured and calculated doses, and less than 3 mm distance to agreement).

As shown in the Bland‐Altman^(4)^ plot (Fig. 4), the mean difference between the expected and absolute measures is 2.3 cGy. The standard deviation (SD) of the difference is 3.26, so the 95% limits of agreement are −6.4 and 11.0 cGy. In Fig. 4, the solid line represents the mean difference and the dashed lines are offset from the mean by ± 1.96 SD. Figure 5 illustrates a QA plan for two arc treatments to a patient's pelvis. Our MapCHECK analysis shows that 98.8% of the points fall within 3% of their expected values and within 3 mm of their expected positions. (In other words, 98.8% of the diodes satisfy the MapCHECK gamma criterion, γ< 1.) This proportion is referred to hereafter as the passing rate. The central axis (CAX) doses provided by MapCHECK for the partial arcs are within 2% of the TPS values.

## IV. DISCUSSION

Quality assurance has been the subject of intense research as a result of advances in planning and delivery techniques. Several methods are being investigated as QA plans for patients undergoing the RA procedure, which can treat many forms of tumor diseases (prostate, pelvis, lung, head/neck, etc.). The core steps of the QA method proposed in this article can be adapted to any RA treatment. First, we compare the absolute doses determined from the TPS to doses measured in our ion chamber phantom. Second, we calculate and compensate for the angular dependence of the dose shape by creating partial arcs and applying them to the MapCHECK phantom. Naturally, the whole QA process must be supplemented by daily patient monitoring, including KV radiograph or CBCT matching, and repeated whenever the treatment plan is modified.

There are few studies comparing absolute doses generated in practice to the dose maps generated by TPS software. Kong and Wang^(5)^ reported 11 prostate cases treated by RA in a Mosaiq environment, using CBCT as a localization device. The average difference between the predicted and measured absolute doses in this sample was 0.47%, with a standard deviation of 1.71% and a passing rate of 97.24% in the dose map.

Some authors have proposed remedying the angular dependence by testing the RA system on a cylindrical phantom surrounded by detectors. An analysis performed by Letourneau et al.,^(6)^ for example, found good agreement between the measured and expected doses in this type of phantom: over 180 control points, more than 86.4% of diodes satisfy the γ< 1 gamma criterion (no more than 3% difference in doses, and 2 mm distance to agreement or DTA). Despite this low percentage, the new version of the phantom accommodated a higher density of diodes and could potentially run at higher energies to treat deep‐seated tumors.

Nicolini et al.^(7)^ used the GLAaS algorithm to assess portal dosimetry for RA quality assurance and extend the QA system to higher energies. They demonstrated that the system could be applied at both low and high energy (6–18 MV). At 6 MV the passing rate was 96.7±1.2% (for the previously defined gamma criterion), while at 18 MV the passing rate was 94.9%. In fact, there is no difference between the two modes of operation because the ranges overlap.

This study describes an RA quality assurance protocol based on the MapCHECK device and an ion chamber phantom. To date, there has been a paucity of data comparing the various approaches and commercially available devices used to validate RA treatment plans. RA is still a novel method, so the literature on QA for RA is sparse. Our technique measures the absolute dose in an ion chamber surrounded by a Solid Water phantom, correcting for the effects of temperature and pressure fluctuations. This procedure results in a smaller relative difference between the TPS prediction and the measured dose than techniques previously described by Kong and Wang^(5)^. In fairness, we note that their work measured the actual dose over a wide range of angles (90°–270°). However, our plane detector has shielding that compromises readings taken near the limits of this range. For this reason, we had to exclude the edge‐on directions (90° or 270°) from our arc experiments. To find a new optimal beam angle for the partial arc, we tested the procedure beforehand at several angles in five‐degree increments. The maximum angle attained without any loss of MLC control points or MLC interlock was 30° for both two arcs and a single arc. Note that Nicolini et al.^(7)^ also used partial arcs, to verify gantry rotation and stability, as well as fluctuations linked to lost composite planes.

Our results demonstrate a strong correlation between the absolute dose predicted by our TPS software and the measured dose. Indeed, there was no significant difference between the two values. The precision of the TPS software was 0.9985, and its accuracy was 0.9992. A Bland‐Altman plot of the results establishes comparable, accurate and reliable confidence limits for RA quality assurance. The results of this study provide a statistically sound description of the absolute dose and dose map.

However, we recognize that our method has several limitations. It is a time‐consuming, two‐step process. In addition, it relies on partial arcs to correct for the angular dependence, and some MLC control points or interlocks are lost when transferring the plan to the system via DICOM. The last problem could be easily overcome by reinitializing the MLC, then choosing the partial arc for the QA plan more carefully via a MLC “detail” function with control points close to 60° and 300°. Perhaps more importantly, we have not yet accounted for all sources of variability in the measurements. While we use two environmental conditions (temperature and pressure) to correct the ion chamber measurements, other factors such as the changing profile of Solid Water buildup material along the beam and the precision of the ion chamber have not been included. Finally, to obtain high‐quality readings, it is crucial that the chamber be fully engaged at the measured point and that all instruments involved be ADCL calibrated.

It is important to bear in mind that in RA treatment planning and delivery, we set the collimator angles to 45° or 315° through the optimizer in order to curtail the tongue and groove effect. In addition, when correcting for the angular dependency of the dose profile, we use partial arcs to determine the shape of the entire plan. In our case, the two arcs (from 60° to 300°) worked perfectly, without any loss of MLC control points and interlock when these plans were uploaded to the machine via DICOM. This success is demonstrated by the excellent agreement between absolute doses, MapCHECK doses on the central axis, and TPS predictions of these doses (the error in the central axis dose is less than 2%). So far, we have not encountered the problem of lost control points as long as we limit our partial arcs to the region of interest and derive them using appropriate control points from the actual plan.

## V. CONCLUSIONS

This study has provided further evidence of the accuracy that can be attained with MapCHECK and an ion chamber phantom. The fluence map calculations were deemed sufficient at the 3% discrepancy level and 3 mm DTA, with a passing rate of 97% for most QA plans studied. The point dose calculations were within 1% of their TPS predictions.

### Appendix

Two phantoms are scanned: one is a 6 cm layer of Solid Water with an embedded ion chamber, and the other is a 3 cm layer of Solid Water placed in front of the MapCHECK detector array. This appendix describes the detailed procedure for testing both QA plans.

**1. Absolute Dose Plan (Phantom 1)**

**Highlight the approved plan:** click on the “Planning” tab and choose “Create a verification plan”.

**Create a QA course:** select Phantom 1 with buildup, then select “Place all fields into the same plan”, then un‐check all boxes, then click on “Finish”.

**Calculate the plan and save it.**

**Export:** Select “File → Export → Wizard” and select the plan. When prompted by the Wizard, choose “Include reference images” and “Compatible with Variant Treatment console”. Next, choose the “Dicom Media Filter” export filter. Finally, change the name of the plan to “Absolute Dose” and save to the local machine. (e.g., N :).

**2. Partial Arc Plan** (Phantom 2)

**Click on** the “Planning” tab and choose “Create a verification plan”.

**Choose** the same QA course. Next, select MapCHECK (Phantom 2). Place all fields into the same plan, un‐check all boxes, and click on “Finish”.

**Calculate the plan and save it.**

**Highlight** this plan by clicking on “Planning”, then “Create partial treatment plan”. Put in the same QA course.

**Change your control points** so that your arc angles are 30° off‐lateral (e.g., 60° and 300°)

**To find the control points** that are closest to these angels, select RMC on the MLC, properties, dynamic, details. Once this is done, click on “Finish”.

**Calculate the plan and save it**.

$1^{\text{st}}$ **Export:** Choose “File → Export → Wizard”, and select the plan. When prompted, select “Include ref images” and “Compatible with variant x console”. Choose the “Dicom Media Filter” export filter. Finally, change the name to Partial Absolute Dose, and save it to the local machine (e.g., N :).

$2^{\text{nd}}$ **Export:** Move the viewing plane to the CAX diode, select RMC on the dose label, export the dose plane, set the X & Y matrix to 22, and click off any burn marker pixels. Then choose MapCHECK, click on “Change objects” to create a new folder for the patient, and save the partial arc plan.
